# Case of Catalepsy Relieved by Music

**Published:** 1846-06

**Authors:** James Bloodgood

**Affiliations:** Cassapolis, Mich.


4. *Case of Catalepsy relieved by Music.*—By James Bloodgood, M. D., of Cassapolis, Mich.

I was called in the evening of Sept. 5th, 1843, to see Dorcas Howard, aged 17, of small stature, and florid complexion, who was said to be in a fit. I found her with a full somewhat accelerated pulse, white tongue, costive bowels, flushed face, and completely cataleptic; the muscles of the eyelids, which I believe is unusual in this rare disease, being affected like all the other muscles of voluntary motion, and with this peculiarity, than when closed a slight impulse communicated to one of them, would cause both to open widely, in which state they would remain until an opposite impulse was given, when both would close simultaneously; but such a balance between the opposing muscles as would leave them partially open after the finger was removed, could not be obtained. Her attending physician, Dr. Allen, of Lagrange, where the case occurred, informed me that she laboured under menstrual suppression, and that the attack was preceded by severe headache. As no notes were taken, the previous treatment was forgotten. We applied cups to the temples, directed a blister to the spine, sinapisms to the extremities, cold applications to the head, and a mixture of jalap and crem. tart. to be kept in the mouth, and which was swallowed involuntarily at intervals through the night.

6th, 9 o'clock. No operation or change in any respect. Having learned that she was extravagantly fond of dancing to the music of a violin, a performer on that instrument was procured, and requested to play one of her favourite tunes, which he did, with immediate and striking effect. Her breathing became hurried and deep, and for a short time she appeared to be making strenuous efforts, like one closely bound, to release herself; she then became quiet, with the exception of the fingers of the right hand, the motions of which corresponded so perfectly with those of the operator's left, as to induce the bystanders to attribute it to mesmerism, which was in high credit here at that time. When the music ceased, she opened her eyes and drank eagerly of water that was presented to her, though still apparently unable to move, and a repetition of the dose, not of water, but of music, restored her to perfect consciousness and volition. Under the operation of a blister to the epigastrium, which was tender, and means to restore the menstrual secretion, she soon recovered, and was subsequently married.

*March* 23, 1845. I was again requested to see her for a similar attack, which had continued five days without medical treatment, the fiddling having been relied on exclusively. The paroxysms were now of an hysterical character, commencing with convulsions, which became frightful if not arrested; but under the operation of the violin, which had been in use almost constantly by night and day, she passed in a few moments from the convulsive to the cataleptic state, and to consciousness as in the first attack, to relapse almost whenever the music ceased. Bleeding, cupping, blistering and cathartics relieved her in a day or two, and she remained as well as could be expected, with the exception of a threatened abortion, for which she was bled, until the 13th Sept. last, when she was delivered of a small healthy child after an easy labour, and has since remained in perfect health. The effect of music in this case was very remarkable. During her sickness she never had a paroxysm which music would not remove, or which was removed without it, though its effect was only temporary until depletory remedies had been used; and those remedies, however necessary they might be to secure a permanent recovery, were never alone sufficient to relieve a paroxysm.—*Ibid.*

